# Shielded‐coaxial‐cable coils as receive and transceive array elements for 7T human MRI

**DOI:** 10.1002/mrm.27964

**Published:** 2019-09-04

**Authors:** Thomas Ruytenberg, Andrew Webb, Irena Zivkovic

**Affiliations:** ^1^ C.J. Gorter Center for High Field MRI Department of Radiology Leiden University Medical Center Leiden The Netherlands

**Keywords:** 7T arrays, coaxial coils, transceive coils

## Abstract

**Purpose:**

To investigate the use of shielded‐coaxial‐cable (SCC) coils as elements for multi‐channel receive‐only and transceive arrays for 7T human MRI and to compare their performance with equivalently sized conventional loop coils.

**Methods:**

The SCC coil element consists of a coaxial loop with interrupted central conductor at the feed‐point side and an interrupted shield at the opposite point. Inter‐element decoupling, transmit efficiency, and sample heating were compared with results from conventional capacitively segmented loop coils. Three multichannel arrays (a 4‐channel receive‐only array and 8‐ and 5‐channel transceive arrays) were constructed. Their inter‐element decoupling was characterized via measured noise correlation matrices and additionally under different flexing conditions of the coils. Thermal measurements were performed and in vivo images were acquired.

**Results:**

The measured and simulated $B_{1}^{+}$ maps of both SCC and conventional loops were very similar. For all the arrays constructed, the inter‐element decoupling was much greater for the SCC elements than the conventional ones. Even under high degrees of flexion, the coupling coefficients were lower than −10 dB, with a much smaller frequency shift than for the conventional coils.

**Conclusion:**

Arrays constructed from SCC elements are mechanically flexible and much less sensitive to changes of the coil shape from circular to elongated than arrays constructed from conventional loop coils, which makes them suitable for construction of size adjustable arrays.

## 1 INTRODUCTION

Transceive and transmit and/or receive arrays are commonly used in ultra‐high field (>3T) MRI because they can be used for B_1_‐shimming^1^, ^2^, ^3^, ^4^, ^5^ as well as accelerated acquisitions using either SENSE or GRAPPA.^6^, ^7^ The major challenge is to minimize the coupling between individual elements of the array, particularly when they are placed close together. Various methods have historically been used including geometrical overlapping,^8^ transformers,^9^ capacitive and/or inductive networks,^10^, ^11^, ^12^, ^13^ and passive resonators.^14^, ^15^, ^16^ Each method can then be combined with impedance mismatching with the preamplifier for an additional ~20–30 dB decoupling.^17^, ^18^ More recently proposed decoupling paradigms include using an uneven distribution of electrical impedances around the length of the loop^19^ and high impedance coils.^20^ The decoupling technique described in Yan et al^19^ is based on the fine tuning of each coil's capacitance distribution to balance magnetic and electric coupling such that they cancel each other. This technique requires a number of lumped elements whose value must be precisely calculated. The decoupling technique proposed in Zhang et al^20^ is essentially based on an inverted pre‐amplifier decoupling^8^ where instead of high impedance at the feeding point (that suppresses current flow), there is a very low impedance at the feeding point. In Zhang et al^20^ the loop is constructed from a coaxial cable with specific characteristic impedance with an interrupted central conductor at the feeding point side and interrupted shield on the opposite side. The authors note in their discussion that 1 limitation of this technique is that the maximum loop size decreases as a function of static field: at 7T, the maximum diameter of the high impedance loop was noted to be 40 mm. A second limitation is that the coils can only be used as receive elements because their decoupling depends on pre‐amplifiers.

In this paper, we investigate the use of shielded‐coaxial‐cable (SCC) coils as elements of transceive and receive arrays. The SCC was first described and used by the amateur radio community.^21^ The SCC has also been described very briefly in the NMR literature. In Harpen,^22^ a mathematic model that describes the resonance spectra of the SCC was developed, although no MR data were presented. In Stensgaard,^23^ the optimization of SCC quality‐factor maximizations for spectroscopic applications at 1.5T was studied, with the authors finding similar performance between the SCC and a conventional loop coil. As such, the SCC is not a new principle in coil design, but its incorporation into transmit or receive arrays has not been described previously, especially with respect to the isolation properties between individual elements of an array. Given its mode of action, it would appear to have several desirable properties in terms of intrinsic isolation. Because of discontinuities in the shield, there is an RF potential difference along its length. In receive mode, the oscillating magnetic field excites current flow on the outer shield wall. Skin effects isolate the outer shield wall from the inner shield wall. The current on the outside of the shield produces a voltage across the open gap in the shield, and this voltage excites current flow on the inner wall of the shield. In turn, the current on the inner wall creates current on the inner conductor through inductive field coupling. The reciprocal mechanism applies in transmission. The shield acts as an antenna whereas the inside of the loop is a simple transformer. Therefore, one can infer that the inter‐element isolation in an array might be considerably better than for a conventional loop coil, which we investigate in this work.

In terms of operation at high frequency for in vivo human use, the resonant frequency is defined by the length of the coaxial cable, which allows circular loops up to 100 mm in diameter to be formed at 7T. It has also been shown in Demaw^24^ and Nohava et al^25^ that introduction of multiple shield gaps or multiple turns can increase and/or decrease the resonant frequency of the SCC coil, so the loop diameter can be adjusted to the desired value for the particular field strength being used. The coaxial cable is flexible, so the coil can conform to the geometry of the body part being imaged, similar to, for example, a liquid metal coil.^26^ Finally, the design can be used in either receive‐only or transceive arrays. The performance of a 4‐element receive‐only array (knee) and 2 transceive arrays of 8 (knee) and 5 (hand) elements were investigated via in vivo imaging of healthy volunteers.

## 2 METHODS

### 2.1 Coil fabrication

**Figure 1:**
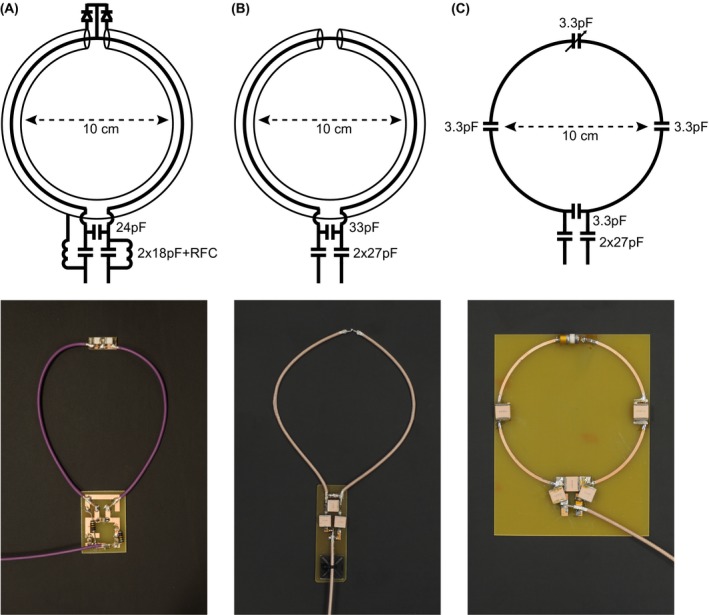
Schematics and photos of (A) receive‐only coaxial loop coil, (B) transceive, and (C) conventional coil

Receive‐only loops (Figure 1A) were made from 1.8 mm diameter flexible coaxial cable (G 01132‐06, Huber+Suhner, Herisau, Switzerland) with a conventional pi‐matching network with 2 18‐pF capacitors (Dalicap, Dalian, China) in the series arms and 1 24‐pF capacitor in the parallel one. An electrically floating copper shield was created on the back of the printed circuit board used for soldering the passive components to shield any stray RF field. Two PIN diodes (MA4P7441F‐1091T, MACOM, Lowell, Massachusetts) were used for detuning purposes and were connected between the inner conductor of the coaxial cable and the shield, at a position opposite from the feed point. The cable shield was interrupted at the top part of the loop (opposite from the feed point), whereas the inner conductor was interrupted at the bottom part. The resonance of the loop is largely determined by the total length of the coaxial cable, as shown in Equations 1 and 2, 24, 25: the resonant condition occurs when the inductive reactance, *X_L_*, is equal to the capacitive reactance, *X_c_*.

(1) $$X_{L}\left(\omega_{0}\right)=\omega_{0}\mu_{0}\frac{d_{0}}{2}\left[\ln\left(\frac{8d_{0}}{d_{1}}\right)-2\right],$$

(2) $$X_{c}\left(\omega_{0}\right)=-Z_{0}\cot\left(\frac{\omega_{0}l\sqrt{\epsilon_{r}}}{c_{0}}\right),$$

where *d*_0_ is the coil diameter, *d*_1_ is the cable diameter, *l* is the stub length between the inner and outer gaps of the coaxial cable, *Z*_0_ is characteristic impedance of cable, and *ɛ*_r_ is permittivity of the dielectric material inside a cable.

For performance comparison purposes, a circular SCC coil with diameter 100 mm was formed, as shown in Figure 1A,B. Conventional circular loops with a diameter of 100 mm were fabricated on an FR‐4 substrate (*ɛ*_r_ = 4.3, tanδ = 0.025, substrate thickness 1.5 mm) with 3 distributed capacitors (3.3 pF, Dalicap), 1 variable tuning capacitor (connected in parallel), and 2 matching capacitors (connected in series, 27 pF), as shown in Figure 1C.

Elliptical transceive loops (120 × 60 mm, Figure 1B) were created using the same design methodology without the PIN diodes. Slightly thicker (3 mm) coaxial cable (K_02252_D‐08, Huber+Suhner) was used for the high transmit power. Two high‐voltage‐rated capacitors of 27‐pF (7200 V, Dalicap) were connected in series, and 1 high‐voltage‐rated 33‐pF capacitor was connected in parallel. The same floating shield was used as described above. For comparison with the transceive array, an elongated loop made of 1 mm copper wire with 4 distributed capacitors (2.2 pF) and 2 matching capacitors (connected in series, 47 pF) was constructed. An 8‐channel annular transmit and/or receive array with elliptical shielded loop coils placed immediately adjacent to one another was designed to cover the full axial extent of the knee. The coils were positioned immediately adjacent to one another. A second transceiver array consisting of 5 coil elements formed on an electrically insulating glove (rated 500 V, GLE36‐00, Regeltex, France) was constructed for imaging the hand. As with the design presented for 3T imaging in Zhang et al.,^20^ 1 coil was placed above each digit.

S‐parameter measurements were performed using a Vector Network Analyzer (TR1300/1, Copper Mountain Technologies, Indianapolis, Indiana) and a rectangular tissue‐mimicking phantom (*ɛ*_r_ = 50 and *σ* = 0.55 S/m, 400 × 400 × 190 mm^3^). A 1‐cm thick foam spacer was placed between the coils and the phantom. The coupling coefficient between 2 loops (SCC and conventional) was measured while varying the amount of overlap from 0 mm to 40 mm. In a second experiment, 3 loops at various inter‐coil separations (25% overlap, immediately adjacent, and spaced by one‐half the loop's diameter) were measured.

The unloaded Q (Q_ul_) and loaded Q (Q_lo_) values of a single conventional and SCC element were measured with a pick‐up coil and a vector network analyzer.

The dependence of the coil's resonant frequency on the coil's geometry was also evaluated for both conventional and SCCs. The coil's geometry was varied from circular (100 mm × 100 mm) to elongated (60 mm × 150 mm) shape and from flat to bent around a 120 mm diameter cylindrical phantom (*ɛ*r = 50 and *σ* = 0.55 S/m).

### 2.2 Electromagnetic simulations

Electromagnetic simulations were performed in CST Microwave Studio 2019 (CST Studio Suite, Computer Simulation Technology, Darmstadt, Germany). Simulations were first carried out with a single antenna element on a square phantom (phantom properties: *ɛ*_r_ = 50 and *σ* = 0.55 S/m) using the frequency domain solver with tetrahedral meshing, as hexahedral meshing is not able to properly mesh a curved coaxial cable. To evaluate the $B_{1}^{+}$ and SAR_10g_ efficiency of the single antenna, results were normalized to 1 W of accepted power. Subsequent in vivo simulations using an 8 element transmit array were performed using the voxel model Gustav (CST Studio Suite, Computer Simulation Technology).

### 2.3 MRI measurements

All MRI measurements were performed on a 7T Philips Achieva scanner, which uses low input impedance preamplifiers with a value of roughly 2 + j5 Ohms.

For phantom experiments, a single transceiver loop and conventional loop were placed 1 cm above the rectangular phantom described previously. $B_{1}^{+}$ maps measured on a phantom were obtained using the dual refocusing echo acquisition mode (DREAM)^27^ sequence with the following parameters: FOV = 400 × 320 × 25 mm^3^, voxel size = 5 × 5 × 5 mm^3^, slices = 5, tip angle = 10°, STEAM angle = 50°, TE/TR = 1.97/15 ms, 1 signal average.

Thermometry measurements were carried out on the same phantom using the transceive elongated SCC and conventional coils. These measurements were carried out using the proton reference frequency method.^28^ A 3D gradient‐echo sequence was used for heating and also for carrying out the thermal measurements^29^: TR/TE = 14/10 ms, flip angle (FA) = 10°, scan duration = 15 min. To induce measurable temperature changes the SAR limits of the scanner were disabled and the power absorbed by the sample was increased by applying a series of 100 kHz off‐resonance pre‐pulses during the imaging sequence (these far off‐resonance pulses do not interfere with the imaging itself). Both coil elements were configured to transmit the same amount of RF power.

To determine how sensitive the coils are to the size of the object being imaged the 4 channel receive‐only array was tested on 3 different sized phantoms with the following circumferences—290 mm, 370 mm, and 410 mm. The coils were tuned for the 370 mm circumference phantom. The individual coil minor axis lengths corresponding to different phantom's circumferences were 73 mm, 93 mm, and 103 mm. Phantom images were obtained with a 3D T_1_‐weighted gradient‐echo sequence using the following parameters: TR/TE = 5.8/2.5 ms, FA = 10°, voxel size = 0.7 × 0.7 × 0.7 mm^3^, number of signal averages (NSA) = 1.

For in vivo experiments all volunteers signed an informed consent form, and the study was approved by the local medical ethics committee. Imaging using the 4‐element receive‐only SCC array was performed on the knee of a healthy volunteer. A quadrature high‐pass birdcage coil (Nova Medical) was used for transmit. In vivo images were obtained with a 3D T_1_‐weighted gradient‐echo sequence using the following parameters: TR/TE = 5.8/2.5 ms, FA = 10°, voxel size = 0.7 × 0.7 × 0.7 mm^3^, NSA = 1.

In vivo measurements using the 8‐element SCC transceive array and 5‐channel glove transceive SCC array were performed using a vendor‐supplied multi‐transmit system. For images of the knee, transmit phases were adjusted for excitation of the CP^+^ mode, and for images of the hand, equal transmit phases were used for all channels. The knee images were obtained on 4 volunteers, with different body mass indices (BMIs), using a 3D T_1_‐weighted gradient‐echo sequence with the following parameters: TR/TE = 5.8/2.5 ms, FA = 10°, voxel size = 0.7 × 0.7 × 0.7 mm^3^, no averaging. The circumferences of the volunteers' knees were 370 mm, 390 mm, 400 mm, and 430 mm. The minor axis lengths of the 8‐channel flexible array elements varied from 46 mm to 54 mm.

The hand images were obtained on 2 volunteers using a 3D T_1_‐weighted gradient‐echo sequence with the following parameters: TR/TE = 25/4.9 ms, FA = 25°, voxel size = 0.5 × 0.5 × 4.0 mm^3^, no averaging, and T_1_w TSE sequence with the following parameters: TR/TE = 23/631 ms, voxel size = 0.5 × 0.5 × 4.0 mm^3^, NSA = 1.

## 3 RESULTS

### 3.1 Comparison of conventional and SCC coils

**Figure 2:**
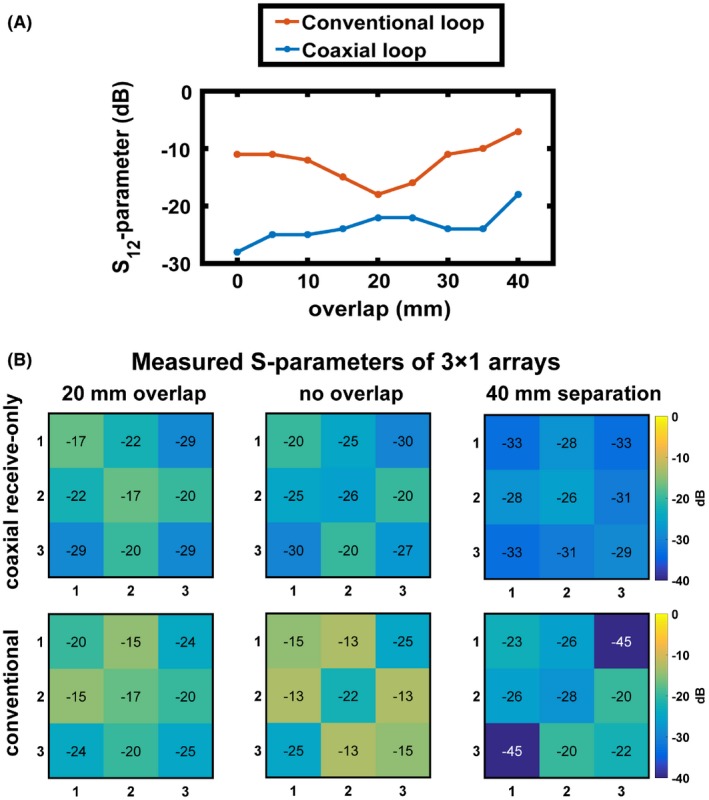
(A) S_12_‐parameters for 2 coils for both the SCC and conventional loop as a function of overlap. The loops have a diameter of 100 mm and were placed on a tissue‐mimicking phantom. (B) Measured S‐parameters of 3 SCCs (top row) and conventional loops (bottom row) placed on the phantom with +20 mm overlap (left column) immediately adjacent to one another (0% overlap, middle column) and 40 mm separation between elements (right column)

Figure 2A depicts the S_12_‐parameters of a 100‐mm diameter circular SCC and an equally sized conventional loop coil as a function of overlap in a 2‐element array placed on the rectangular phantom. Although the conventional loops show minimum coupling at ~20 mm overlap as expected, the SCC loops have no sharp optimum value and have a higher decoupling for every degree of overlap/separation. To investigate next‐neighbor coupling, Figure 2B shows the measured S‐parameter matrix of 3 elements of circular SCCs and its conventional analogues. The measured inter‐element coupling was, in general, lower for the SCCs compared to the conventional surface coils.

**Figure 3:**
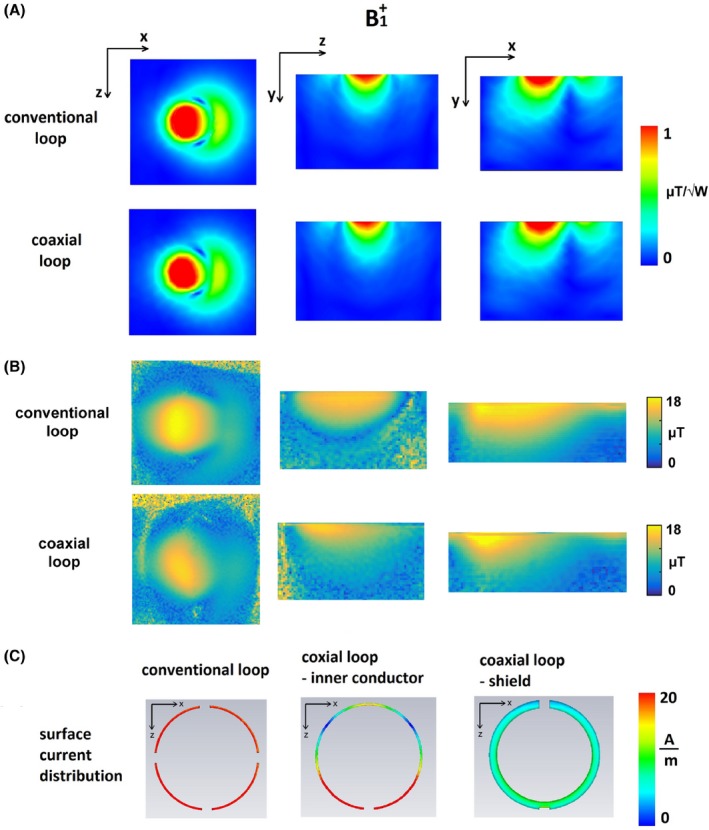
(A) Simulated $B_{1}^{+}$ distributions of conventional and coaxial loops, normalized to 1 W of accepted power. (B) Measured $B_{1}^{+}$ distributions of conventional and coaxial loops. (C) Surface current distributions on conductor of conventional loop (left), inner conductor of SCC (middle), and on shield of SCC (right)

Figure 3 shows simulated and measured $B_{1}^{+}$ distributions and simulated surface current distributions on circular conventional and SCCs. Simulated and measured $B_{1}^{+}$ distributions are very similar for both SCC and conventional coil. There was around 15% lower $B_{1}^{+}$ efficiency of the SCC at superficial depths, whereas at depths of ~50 mm and higher, the efficiencies were comparable. The simulated surface current distributions show evenly distributed surface current magnitude on the conventional coil, while on the inner part of coaxial coil the surface current has its maximum at the bottom part of loop (around feeding point) and has variable magnitude at the top part (around shield gap point). The magnitude of the surface current distribution on the shield of the coaxial coil was almost 1 order of magnitude lower than the magnitude of the surface current on the conventional coil.

**Figure 4:**
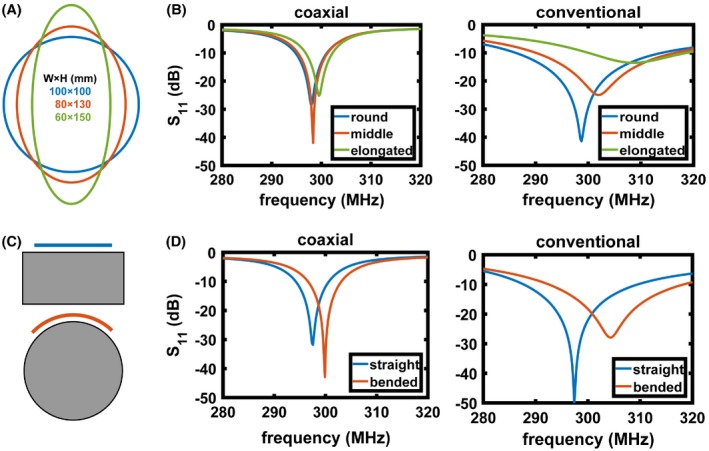
(A) Degree of elongation of the loops, starting from circular (diameter 100 mm), middle elongated (diameter 80 mm), and elongated (diameter 60 mm). (B) Measured S_11_ of SCC (left) and conventional (right) coils when the shape was changed from circular to elongated. The coils were initially tuned at circular shape. (C) Schematics of the coil position on a flat and cylindrical phantom. (D) Measured S_11_ of SCC (left) and conventional (right) coils when placed on a flat phantom and bent on a cylindrical phantom. The coils were initially tuned when placed on a flat phantom

Figure 4 shows the measured resonant frequency shift when the coil geometry is changed from circular (100 mm × 100 mm) to slightly elongated (80 mm × 130 mm) to elongated (60 mm × 150 mm). The coils were initially tuned to resonate at 298 MHz for the circular geometry. The shifts in resonant frequency for the conventional coil were 2.1 MHz and 11.1 MHz (Figure 4B), respectively, whereas those for the SCC were 0.3 MHz and 1.5 MHz, respectively. Figure 4C,D show the resonant frequency shifts when the coils were bent around the cylindrical phantom. The resonant frequency shift of the conventional coil was 7 MHz whereas that of the SCC was 2.5 MHz.

Measured unloaded (Q_ul_)/loaded (Q_lo_) Q factors of the conventional coil were 105/20 whereas those of the SCC were 100/60, corresponding to Q_ul_/Q_lo_ ratios of 5.3 and 1.7.

### 3.2 Receive only array—knee imaging

**Figure 5:**
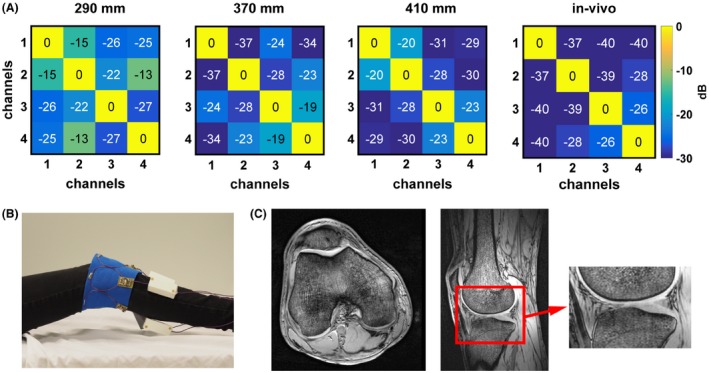
(A) Measured noise correlation matrices of the 4 channel receive array on phantoms of the following circumferences—290 mm, 370 mm, and 410 mm. The coils were tuned on a phantom with a circumference of 370 mm. Measured in vivo noise correlation matrix. (B) Photograph of the in vivo measurement setup consisting of 4 non‐overlapped receive loops (a birdcage coil was used for transmit and is not shown on the image). (C) In vivo images of the knee. A magnified image of the cartilage is shown to demonstrate the fine structure.

Figure 5A shows measured noise correlation matrices of the 4 channel receive‐only SCC array on 3 different phantom circumferences—290 mm (coil's minor axis length was 73 mm), 370 mm (coil's minor axis length was 93 mm), and 410 mm (loop diameter was 103 mm). The S_11_ of individual channels was tuned on the phantom with 370 mm circumference. The highest measured coupling between the channels on a phantom with 290 mm circumference was −13 dB (S_11_ of individual channels of this array was −13 dB or better). The highest measured coupling between the channels on a phantom with 370 mm circumference was −19 dB (S_11_ of individual channels of this array was −20 dB or better). The highest measured coupling between the channels on a phantom with 410 mm circumference was −20 dB (S_11_ of individual channels of this array was −15 dB or better). The coil coupling between the elements in the in vivo measurement was lower than −26 dB (S11 of individual channels was −20 dB or better). Figure 5B shows the measurement setup with 4 receive‐only loops placed around the knee of a volunteer. The loops were placed immediately adjacent to one another. High resolution gradient‐echo images were obtained in both sagittal and axial orientations using an isotropic voxel size of 0.7 mm^3^ demonstrating excellent visualization of the cartilage and trabecular bone structure.

### 3.3 Transceive array – knee imaging

**Figure 6:**
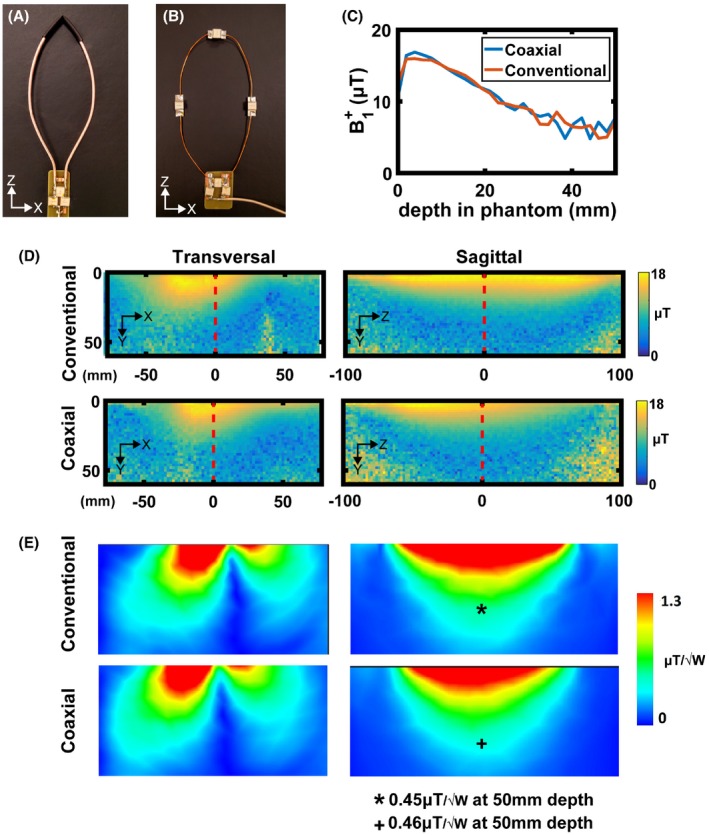
Photographs of a (A) SCC elongated loop and (B) conventional elongated loop. (C) Measured $B_{1}^{+}$ profile along the central axis of the antenna (red dashed line in D). (D) Measured transversal and sagittal $B_{1}^{+}$ maps of conventional and SCC coils. (E) Simulated transversal and sagittal $B_{1}^{+}$ maps of conventional and SCC coils

For a transceive array for knee imaging, we constructed an 8 element SCC array, and in order for these to be accommodated around the knee, the coils need to be elongated. Figure 6A,B show photographs of the elongated SCC and conventional coils, respectively. Figure 6D,E show measured and simulated $B_{1}^{+}$ maps of a single elliptical transceiver SCC compared to the conventional elongated loop coil segmented by 4 capacitors. Both loops were placed on the same phantom and imaged at the same time using individual channels of the multiple transmit setup to ensure that the same input power and imaging conditions are used. Both transverse and sagittal profiles are shown. The $B_{1}^{+}$ maps show very similar intensity profiles for the SCC and conventional loops, although it should be noted that the SCC acts as a slightly (~8%) “shorter coil” in the sagittal plane. Figure 6C shows $B_{1}^{+}$ profiles of SCC and conventional coils plotted along the central lines (red dashed line in Figure 6D). The $B_{1}^{+}$ efficiency of the conventional coil is slightly better than the efficiency of SCC at superficial depths. At depths ~50 mm and more, the efficiency of the SCC becomes comparable or better than the efficiency of the conventional coil. Supporting Information Figure S1 shows the measured S‐parameter matrices of 3 elements of the elongated transceive loops placed on the rectangular phantom with +20 mm overlap, immediately adjacent to one another and 40 mm separation between coils. High levels of inter‐element decoupling were achieved for each case.

**Figure 7:**
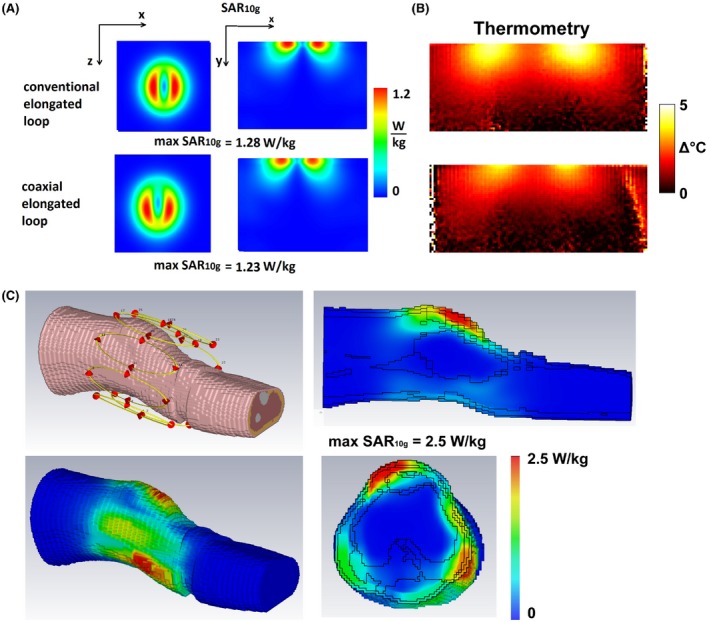
(A) Simulated SAR_10g_ of a single element on a phantom of conventional elongated (upper image) and coaxial elongated (lower image) coil. (B) Corresponding thermometry measurements of elongated conventional and coaxial coils, normalized to the maximum temperature. (C) Simulated SAR_10g_ on a voxel model

Figure 7A shows simulated max SAR_10g_ of the elongated conventional and SCC coils. The results are very similar—maximum SAR_10g_ of conventional coil was 1.28 W/kg and of SCC was 1.23 W/kg. Thermometry data of the conventional and SCCs are shown in Figure 7B. These data was corrected for any B_0_ drift during the experiment. The acquired thermometry maps were normalized to the maximum temperature increase. The maximum temperature produced by the conventional coil was around 12% higher than the temperature produced by the SCC. Figure 7C shows maximum SAR_10g_ simulated by 8‐channel array of elongated coils placed around a knee of a voxel model. The maximum simulated SAR_10g_ was 2.5 W/kg.

**Figure 8:**
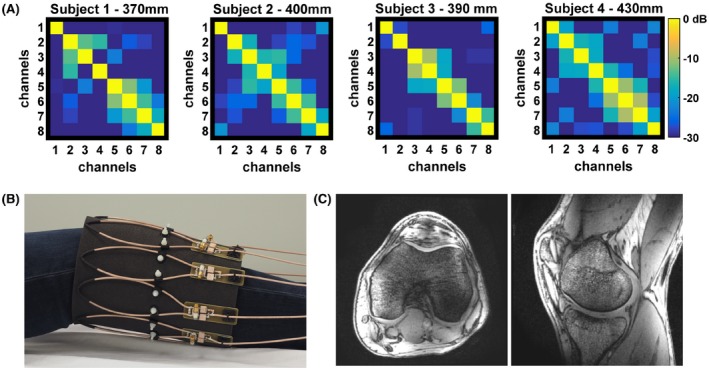
(A) Measured noise correlation matrices on 4 different subjects with different knee circumferences. (B) Photograph of the in vivo measurement setup consisting of 8 non‐overlapped transceive loops. (C) In vivo images of the knee

Figure 8A shows measured noise correlation matrices of the 8‐channel SCC array placed around the knee for 4 different volunteers. The knee circumferences varied from 370 mm to 430 mm. The flexed single coil element minor axis length varied from 46 mm to 54 mm. The highest coupling coefficient was measured in subjects 3 and 4 and was −10 dB. The other coupling coefficients were −14 dB and better.

Figure 8B shows a photograph of the 8‐channel elongated transceive array placed around the knee of a healthy volunteer. Both sagittal and axial images are shown (Figure 8C). Some shading at the centre of the image is evident in the images for which no post‐processing correction has been applied.

### 3.4 Transceive array—hand imaging

**Figure 9:**
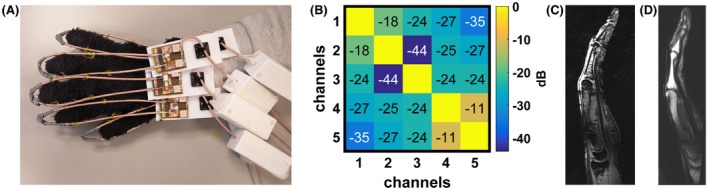
(A) Photograph of the in vivo measurement setup consisting of 5 transceive loops attached to a glove. (B) In vivo measured noise correlation matrix. In vivo images of the hand in natural position showing a single finger in sagittal view using a (C) gradient and (D) spin echo sequence

Figure 9 shows results from the 5‐channel glove transceiver array. The highest inter‐element coupling was between elements 4 and 5 (−11 dB). Different hand sizes did not change the coil's loading significantly. Figure 9C,D show fine bone structure visible on a single finger image with both gradient and spin echo sequences. S_11_ parameters were measured also for different flexion angles of the glove (results not included), and the worst case showed only a 1–2 dB higher S_11_ value at maximum flexion.

## 4 DISCUSSION

This paper has demonstrated a simple method for constructing loop arrays with a high degree of inter‐element decoupling using SCCs. In this design, distributed lumped elements within the loop are not required. The concept is very similar to designs proposed in Zhang et al,^20^ Demaw,^21^ Harpen,^22^ and Stensgaard^23^ for low frequency amateur radio communications and to the cross‐over coil shown by Mispelter et al^30^ that has a slightly different configuration in which the inner conductors in the cross‐over coil are cross‐connected to the shield at the shield gap.

As shown by Avdievich et al.,^31^ the magnetic coupling coefficient (k_m_) of 2 loops (~100 mm diameter) at 300 MHz is very low. The dominant coupling is therefore resistive (k_e_) and occurs from coil‐to‐coil through the sample. Because the ratio of the unloaded‐to‐loaded Q factor of the SCC is more than 3 times lower than that of the conventional coil (Q_ul_/Q_lo_ of conventional coil was 5.3 and of SCC was 1.7), this implies lower coupling to the sample and lower inter‐element coupling because of a lower k_e_. This can further be explained as follows. The inductive E‐field (produced by surface currents on the coil) induces eddy currents in the sample. Induced eddy currents in the sample produce a secondary E‐field that induces currents back to the coil. The stronger the surface currents on the coil, the stronger the coupling (eddy currents) to the sample, which reflects as a lower Q‐factor of the loaded conventional coil. The shield of the coaxial coil partially “shields” the surface currents on the inner conductor, such that the coupling to the sample is lower than the coupling of the conventional coil.

From the surface current distribution shown in Figure 3, the lowest current magnitude is on the shield of SCC (almost 1 order of magnitude lower than the surface current on conventional coil). The surface current distribution on inner conductor of the SCC and on conductor of conventional coil are of similar magnitude. Because the inner conductor of the SCC does not have distributed capacitors, wave effects are noticeable in the magnitude of the surface current.

From $B_{1}^{+}$ simulations and measurements, it can be concluded that the conventional coil is more efficient element at superficial depths, whereas at depths of ~50 mm and more, the efficiency of SCC becomes comparable or better than that of the conventional coil. The general $B_{1}^{+}$ distributions of both conventional and SCC show similar patterns, although the longitudinal $B_{1}^{+}$ coverage in the sagittal plane of the conventional coil is larger than the coverage of the SCC because of the lower current densities on a shield closer to the shield gap and on inner conductor of the SCC. From SAR_10g_ simulations and thermometry measurements, it can also be concluded that an SCC and conventional coil induce similar electric fields in a conducting sample.

Results also show that changing the shape from round to elongated or bending the coil element has a much smaller effect on the tuning and matching of the SCC than for the conventional coil.

## 5 CONCLUSION

Receive and transceive arrays of SCC elements showed a higher degree of intrinsic inter‐element decoupling than conventional loops. This allows the simple construction of flexible multi‐element arrays for high field MRI. The SCC can be used in a receive‐only or in a transceive array. It should be possible to expand the application of the proposed coil concept for imaging body parts for which rigid coil design is not suitable, such as the larynx.^32^ The SCC is also potentially useful as an array element in size‐adjustable tight‐fitting head arrays.

## Supporting information


**FIGURE S1** Measured S‐parameters of 3 transceive coaxial elongated loops placed on a rectangular phantom with +20 mm overlap (left), immediately adjacent to one another (0% overlap, middle), and 40 mm separation between coils (right)

## References

[1] Mao W , Smith MB , Collins CM . Exploring the limits of RF shimming for high‐field MRI of the human head. Magn Reson Med. 2006;56:918–922. 16958070 10.1002/mrm.21013 PMC4040521

[2] Abraham R , Ibrahim TS . Proposed radiofrequency phased‐array excitation scheme for homogenous and localized 7‐Tesla whole‐body imaging based on full‐wave numerical simulations. Magn Reson Med. 2007;57:235–242. 17260366 10.1002/mrm.21139

[3] Vaughan JT , Adriany G , Snyder CJ , et al. Efficient high‐frequency body coil for high‐field MRI. Magn Reson Med. 2004;52:851–859. 15389967 10.1002/mrm.20177

[4] Graessl A , Renz W , Hezel F , et al. Modular 32‐channel transciever coil array for cardiac MRI at 7.0T. Magn Reson Med. 2014;72:276–290. 23904404 10.1002/mrm.24903

[5] Adriany G , Van de Moortele P‐F , Wiesinger F , et al. Transmit and receive transmission line arrays for 7 Tesla parallel imaging. Magn Reson Med. 2005;53:434–445. 15678527 10.1002/mrm.20321

[6] Pruessmann KP , Weiger M , Scheidegger MB , Boesiger P . SENSE: sensitivity encoding for fast MRI. Magn Reson Med. 1999;42:952–962. 10542355

[7] Griswold MA , Jakob PM , Heidemann RM , et al. Generalized autocalibrating partially parallel acquisitions (GRAPPA). Magn Reson Med. 2002;47:1202–1210. 12111967 10.1002/mrm.10171

[8] Roemer PB , Edelstein WA , Hayes CE , Souza SP , Mueller OM . The NMR phased array. Magn Reson Med. 1990;16:192–225. 2266841 10.1002/mrm.1910160203

[9] Shajan G , Kozlov M , Hoffmann J , Turner R , Scheffler K , Pohmann R . A 16‐channel dual‐row transmit array in combination with a 31‐element receive array for human brain imaging at 9.4 T. Magn Reson Med. 2014;71:870–879. 23483645 10.1002/mrm.24726

[10] Zhang XZ , Webb A . Design of a capacitively decoupled transmit/receive NMR phased array for high field microscopy at 14.1 T. J Magn Reson. 2004;170:149–155. 15324768 10.1016/j.jmr.2004.05.004

[11] Bilgen M . Inductively‐overcoupled coil design for high resolution magnetic resonance imaging. Biomed Eng Online. 2006;5:3. 16401343 10.1186/1475-925X-5-3 PMC1363722

[12] Lee RF , Giaquinto RO , Hardy CJ . Coupling and decoupling theory and its application to the MRI phased array. Magn Reson Med. 2002;48:203–213. 12111947 10.1002/mrm.10186

[13] Wu B , Zhang X , Qu P , Shen G . Design of an inductively decoupled microstrip array at 9.4T. J Magn Reson. 2006;182:126–132. 16829145 10.1016/j.jmr.2006.04.013

[14] Avdievich NI , Pan JW , Hetherington HP . Resonant inductive decoupling (RID) for transceiver arrays to compensate for both reactive and resistive components of the mutual impedance. NMR Biomed. 2013;26:1547–1554. 23775840 10.1002/nbm.2989 PMC3800502

[15] Yan XQ , Zhang XL , Feng BT , Ma CX , Wei L , Xue R . 7T transmit/receive arrays using ICE decoupling for human head MR imaging. IEEE Trans Med Imaging. 2014;33:1781–1787. 24710826 10.1109/TMI.2014.2313879

[16] Connell I , Gilbert KM , Abou‐Khousa MA , Menon RS . Design of a parallel transmit head coil at 7T with magnetic wall distributed filters. IEEE Trans Med Imaging. 2015;34:836–845. 25415982 10.1109/TMI.2014.2370533

[17] Keil B , Blau JN , Biber S , et al. A 64‐channel 3T array coil for accelerated brain MRI. Magn Reson Med. 2013;70:248–258. 22851312 10.1002/mrm.24427 PMC3538896

[18] de Zwart JA , Ledden PJ , Kellman P , van Gelderen P , Duyn JH . Design of a SENSE‐optimized high‐sensitivity MRI receive coil for brain imaging. Magn Reson Med. 2002;47:1218–1227. 12111969 10.1002/mrm.10169

[19] Yan X , Gore JC , Grissom WA . Self‐decoupled radiofrequency coils for magnetic resonance imaging. Nat Commun. 2018;9:3481. 30154408 10.1038/s41467-018-05585-8 PMC6113296

[20] Zhang B , Sodickson DK , Cloos MA . A high‐impedance detector‐array glove for magnetic resonance imaging of the hand. Nat Biomed Eng. 2018;2:570–577. 30854251 10.1038/s41551-018-0233-y PMC6405230

[21] Demaw D . On ground low‐noise receiving antennas. QST. 1988;4:30–32.

[22] Harpen MD . The theory of shielded loop resonators. Magn Reson Med. 1994;32:785–788. 7869902 10.1002/mrm.1910320615

[23] Stensgaard A . Optimized design of the shielded‐loop resonator. J Magn Reson A. 1996;122:120–125. 10.1006/jmre.1996.1103 9245363

[24] Nohava L , Czerny R , Obermann M , et al. Flexible multi‐turn multi‐gap coaxial RF coils (MTMG‐CCs): design concept and bench validation. In Proceedings of the 27th Annual Meeting of ISMRM, Montreal, Canada, 2019 p. 0565.

[25] Czerny R , Nohava L , Frass‐Kriegl R , Felblinger J , Ginefri J , Laistler E . Flexible multi‐turn multi‐gap coaxial RF coils: enabling a large range of coil sizes. In Proceedings of the 27th Annual Meeting of ISMRM, Montreal, Canada, 2019 p. 1550.

[26] Port A , Albisetti L , Varga A , et al. Liquid metal in stretchable tubes: a wearable 4‐channel knee array. In Proceedings of the 27th Annual Meeting of ISMRM, Montreal, Canada, 2019 p. 1114.

[27] Nehrke K , Bornert P . DREAM ‐ a novel approach for robust, ultrafast, multislice B‐1 mapping. Magn Reson Med. 2012;68:1517–1526. 22252850 10.1002/mrm.24158

[28] De Poorter J , De Wagter C , De Deene Y , Thomsen C , Stahlberg F , Achten E . Noninvasive MRI thermometry with the proton resonance frequency (PRF) method: in vivo results in human muscle. Magn Reson Med. 1995;33:74–81. 7891538 10.1002/mrm.1910330111

[29] Brink W , Wu Z , Webb A . A simple head‐sized phantom for realistic static and radiofrequency characterization at high fields. Magn Reson Med. 2018;80:1738–1745. 29498102 10.1002/mrm.27153

[30] Mispelter J , Lupu M , Briguet A . NMR probeheads for biphysical and biomedical experiments. London: Imperial College Press; 2006:596.

[31] Avdievich NI , Pfrommer A , Giapitzakis IA , Henning A . Analytical modeling provides new insight into complex mutual coupling between surface loops at ultrahigh fields. NMR Biomed. 2017;30:e3759. 10.1002/nbm.3759 28632306

[32] Ruytenberg T , Verbist BM , Vonk‐Van Oosten J , Astreinidou E , Sjogren EV , Webb AG . Improvements in high resolution laryngeal magnetic resonance imaging for preoperative transoral laser microsurgery and radiotherapy considerations in early lesions. Front Oncol. 2018;8:1–8. 29928638 10.3389/fonc.2018.00216 PMC5997776

