# Supplementary material for: Shielded‐coaxial‐cable coils as receive and transceive array elements for 7T human MRI
Source: Magn Reson Med. 2019 Sep 4;83(3):1135–46. doi: 10.1002/mrm.27964 (PMC6899981; doi:10.1002/mrm.27964)
Supplement: Supplementary file 1 — FIGURE S1 Measured S‐parameters of 3 transceive coaxial elongated loops placed on a rectangular phantom with +20 mm overlap (left), immediately adjacent to one another (0% overlap, middle), and 40 mm separation between coils (right) [file MRM-83-1135-s001.docx]

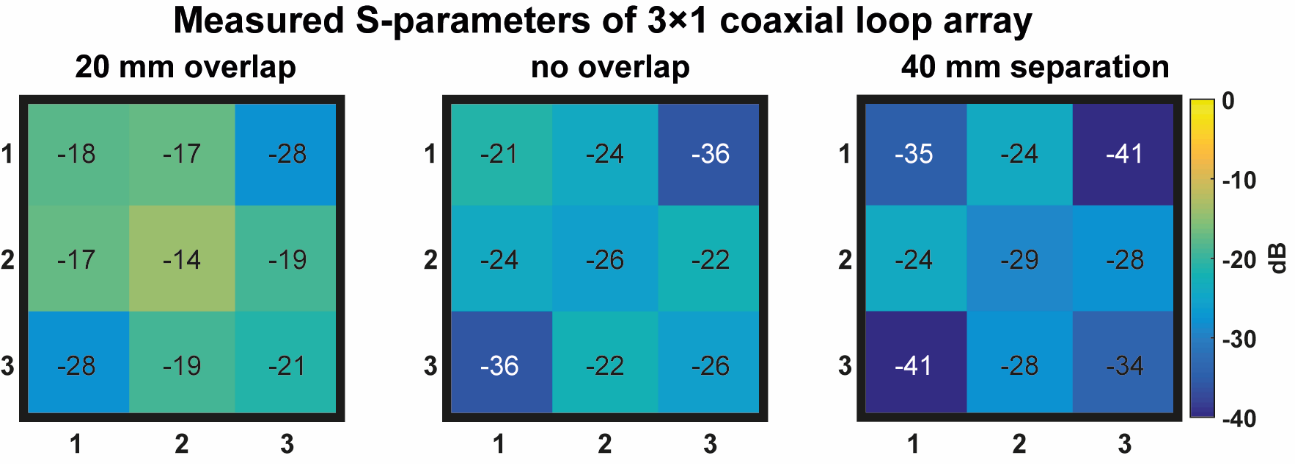


Supplementary Figure S1. Measured S-parameters of three transcieve coaxial elongated loops placed on a rectangular phantom with +20 mm overlap (left), immediately adjacent to one another (0% overlap, middle) and 40 mm separation between coils (right).
